# Comparison of the PIPAS severity score tool and the QSOFA criteria for predicting in-hospital mortality of peritonitis in a tertiary hospital in Uganda: a prospective cohort study

**DOI:** 10.1186/s12893-022-01743-4

**Published:** 2022-07-28

**Authors:** Richard Newton Iranya, Ronald Mbiine, Andrew Weil Semulimi, Joan Nasige, Timothy Makumbi, Moses Galukande

**Affiliations:** 1 Department of Surgery, School of Medicine, Makerere University, Kampala, Uganda; 2 Department of Physiology, School of Biomedical Sciences, Makerere University, Kampala, Uganda

**Keywords:** PIPAS, qSOFA, Peritonitis, Mortality

## Abstract

**Background:**

The majority of the prognostic scoring tools for peritonitis are impractical in low resource settings because they are complex while others are quite costly. The quick Sepsis-related Organ Failure Assessment (qSOFA) score and the Physiologic Indicators for Prognosis in Abdominal Sepsis (PIPAS) severity score are two strictly bedside prognostic tools but their predictive ability for mortality of peritonitis is yet to be compared. We compared the predictive ability of the qSOFA criteria and the PIPAS severity score for in-hospital mortality of peritonitis.

**Method:**

This was a prospective cohort study on consecutive peritonitis cases managed surgically in a tertiary hospital in Uganda between October 2020 to June 2021. PIPAS severity score and qSOFA score were assessed preoperatively for each case and all cases were then followed up intra- and postoperatively until discharge from the hospital, or up to 30 days if the in-hospital stay was prolonged; the outcome of interest was in-hospital mortality. We used Receiver Operating Characteristic curve analysis to assess and compare the predictive abilities of these two tools for peritonitis in-hospital mortality. All tests were 2 sided (p < 0.05) with 95% confidence intervals.

**Results:**

We evaluated 136 peritonitis cases. Their mean age was 34.4 years (standard deviation = 14.5). The male to female ratio was 3:1. The overall in-hospital mortality rate for peritonitis was 12.5%. The PIPAS severity score had a significantly better discriminative ability (AUC = 0.893, 95% CI 0.801–0.986) than the qSOFA score (AUC = 0.770, 95% CI 0.620–0.920) for peritonitis mortality (p = 0.0443). The best PIPAS severity cut-off score (a score of > = 2) had sensitivity and specificity of 76.5%, and 93.3% respectively, while the corresponding values for the qSOFA criteria (score > = 2), were 58.8% and 98.3% respectively.

**Conclusions:**

The in-hospital mortality in this cohort of peritonitis cases was high. The PIPAS severity score tool has a superior predictive ability and higher sensitivity for peritonitis in-hospital mortality than the qSOFA score tool although the latter tool is more specific. We recommend the use of the PIPAS severity score as the initial prognostic tool for peritonitis cases in the emergency department.

**Supplementary Information:**

The online version contains supplementary material available at 10.1186/s12893-022-01743-4.

## Introduction

The mortality associated with delayed or inappropriate initial management of peritonitis cases is unacceptably high worldwide. Even in state-of-the-art surgical emergency centers, mortality rates of up to 40% have been reported among peritonitis cases with established sepsis and septic shock [1–6].

In the Sub-Saharan African region where most surgical centers have glaring healthcare resource gaps (namely: human resources, theatre space, and supplies as well as ICU space), outcomes of peritonitis are suboptimal, and saving especially those cases with advanced physiologic derangement is an enormous task [7–9]. In this setting, therefore, the importance of early prognostic risk stratification to prioritize those at most risk of death for a prompt aggressive therapeutic approach cannot be overemphasized [10].

Several prognostic scoring systems both specific and nonspecific have been validated for use in peritonitis such as the Mannheim Peritonitis Index (MPI) [11], The World Society of Emergency Surgery Sepsis Severity Score (WSESSSS) [12], the Predisposition, Infection Response Organ dysfunction score for Intra-Abdominal sepsis (PIRO-IAS) [13] and the APACHE II score [14–16]. These tools, however, are not strictly preoperative, and/or bedside. Moreover, some require laboratory parameters that are not easily obtainable in resource-limited surgical emergency centers. These tools, therefore, play a limited role in early preoperative risk stratification as well as therapeutic decision-making for individual peritonitis patients. They have instead found roles in research and surgical audits [2].

The Sepsis 3 task force in 2016 recommended the use of the quick Sequential (Sepsis-related) Organ Function Assessment (qSOFA) criteria for quick, bedside triage of septic patients at risk of mortality [17], and indeed many surgical emergency centers in Uganda have since adopted this tool for early prognostication of sepsis-related conditions including peritonitis but this practice has not been backed by robust local evidence. Several observational studies in other centers have reported the qSOFA criteria to be specific but lack adequate sensitivity for identifying septic patients at risk of poor outcomes [18–21]. Tolonen and colleagues in a study to evaluate the predictive abilities of several prognostic scoring systems for peritonitis outcomes found the qSOFA criteria to have a high specificity (95%) but very low sensitivity (37%) for peritonitis mortality [22].

Recently (2019), The world society of emergency surgery (WSES) designed the Physiologic Indicators for Prognosis in Abdominal Sepsis-score (PIPAS severity score) as a bedside early prognostic scoring tool for peritonitis mortality [10]. The tool has 8 variables obtainable from history and physical examination. Each variable is scored 0 or 1 with a maximum score of 8, the higher the score the worse the outcome. Sartelli and colleagues found the PIPAS severity score to have a good predictive ability for mortality of peritonitis in a mainly European cohort [10]. The performance of this tool is yet to be evaluated in our setting.

The qSOFA criteria and the PIPAS severity score tools are both easy to assess, strictly bedside, and have solely preoperative parameters, hence, have the potential to be practically useful for early prognostic evaluation for peritonitis but the predictive ability of these tools for in-hospital mortality of peritonitis in our setting is yet to be assessed and compared. We set out to assess and compare the discriminative ability of the PIPAS severity score and the qSOFA criteria for in-hospital mortality of peritonitis in a resource-limited tertiary center in Uganda.

## Method

### The study design and setting

We conducted a prospective cohort study on peritonitis cases admitted to the surgical unit of Mulago National Referral Hospital (MNRH) for 9 months from 1st October 2020 to 31st of June 2021. MNRH is an 1800-bed capacity hospital located in Kampala, the capital city of Uganda. It is one of the national referral hospitals and doubles as the teaching hospital for Makerere university’s college of health sciences. The hospital’s accident and emergency (A&E) unit is an initial entry point for all trauma and non-trauma surgical emergencies. Here clinically suspected peritonitis cases are triaged, and those needing emergency surgical intervention are stabilized and then operated on before transfer to various surgical wards or to the ICU where appropriate for post-operative care and/or a further planned intervention or re-intervention.

### The study population and sampling

We consecutively recruited patients who were 13 years or older, with the clinical diagnosis of peritonitis and admitted to the surgical unit of MNRH. A case of peritonitis was defined as clinical symptoms and signs (abdominal tenderness, guarding, and/or rigidity with or without imaging signs) suggestive of peritonitis, and evidence of peritoneal contamination confirmed intraoperatively by the primary surgeon. We excluded trauma patients who had peritonitis and concurrent major injuries to other regions of the body because of the likelihood of the latter having a significant impact on mortality outcome. Suspected peritonitis cases that were managed conservatively were also excluded as this group did not fully satisfy our case definition. The sample size for the study was estimated based on the formula suggested by Hajian-Tilaki (2014) for studies comparing the accuracy of two prognostic/diagnostic tools on the same study subjects [23]. We used the AUC for the PIPAS severity score from the previous study [10]. Assuming a detection of an effect size d = (AUC_1_-AUC_2_) of 10% with 95% confidence level and 80% power gave us a sample size of 91, we adjusted upwards of 15% for non-response or loss to follow-up cases to give us a sample size of approximately 136 study participants.

### Study procedures and data collection

All study participants underwent the hospital’s routine initial resuscitation protocol for peritonitis which included: The correction of fluid and electrolyte deficits using intravenous fluids, nasogastric tube suctioning, urethral catheterization, and administration of broad-spectrum antibiotic therapy within 1 h of admission.

The assessment for the PIPAS severity score [10] and the qSOFA score [17] variables for each study participant was done after the initial resuscitation (within 1 h before anesthesia induction). These variables were: Age (in completed years), pre-existing medical conditions (i.e., history of Severe chronic kidney disease, severe cardiovascular disease, and malignancy), respiratory rate (breaths/minute), blood pressure (mmHg), peripheral blood oxygen saturation level (SpO_2_) in room air (percentages), and level of consciousness using both the AVPU (Alert/Verbal/Pain/Unresponsive) responsiveness scale and Glasgow Coma Scale (GCS). In addition, the sex of the study participant was also recorded.

Each case was followed up intraoperatively and then daily thereafter until their discharge from the hospital, or up to 30 days if in-hospital admission was longer than 30 days. In the follow-up evaluation, the data elements recorded for each study participant were: The time interval from admission to definitive intervention (laparotomy), the Source of peritoneal contamination (anatomic site), the need for reoperation (re-laparotomy), and the outcome at discharge (survivor or non-survivor). Those with in-hospital admission longer than 30 days were categorized as survivors.

### Data management and analysis

All study data were entered in Epidata version 4.6 software (Epidata Association, Odense, Denmark), cleaned, and then exported to STATA version 16 for analysis.

Descriptive statistics were used to characterize our study population; continuous normally distributed variables were summarized into mean and standard deviation and in median and interquartile range if non-normally distributed, while categorical data were summarized into absolute frequencies, proportions, and percentages.

We used chi-square statistics or t-test to compare baseline characteristics among survivors and non-survivors of peritonitis cases. Binary logistic regression models PIPAS and qSOFA scores were created for predicting in-hospital mortality of peritonitis. Each model’s suitability was assessed using the Hosmer-Lemeshow goodness of fit test.

The discriminative ability of the two tools (i.e., the PIPAS severity score and the qSOFA score tools) for in-hospital mortality of peritonitis were assessed using a non-parametric estimate of the Area Under the receiver operating characteristic Curves (AUC) with bootstrap resampling inference. The AUC of the tools was then compared using Delong’s method [24]. The optimum cut-off score (for each tool) for predicting in-hospital mortality of peritonitis was determined using Youden’s index. All statistical analyses were 2-sided (p < 0.05), with 95% confidence intervals using STATA version 16 (StataCorp, College Station, Texas, USA).

## Results

### Baseline characteristics of the study population

**Fig. 1:**
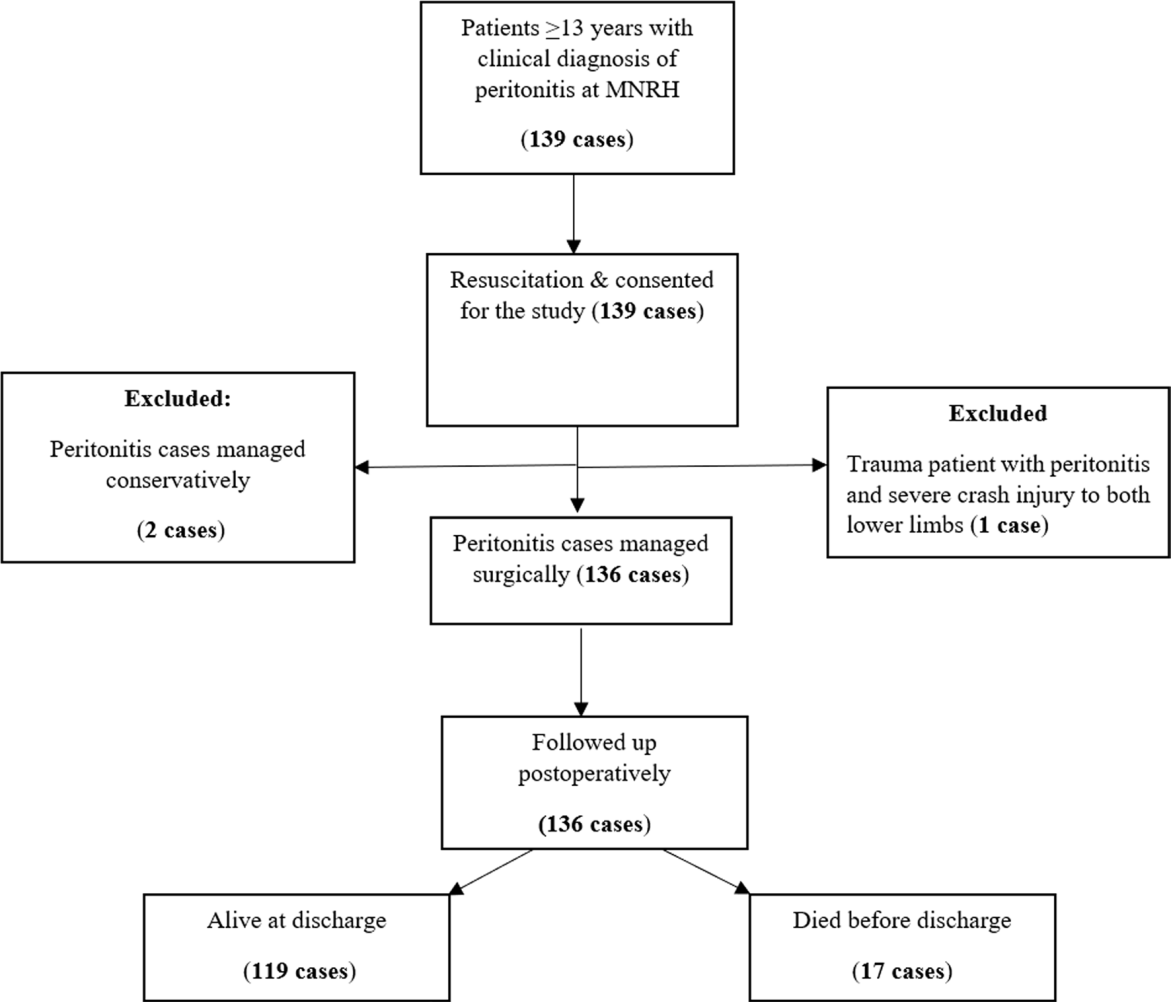
Flow-chart showing recruitment of study participants into the study

A total of 139 peritonitis patients were assessed, and 136 cases met the inclusion criteria for the study. The 3 cases excluded were (two clinically suspected peritonitis cases that were managed conservatively and one multiply injured patient with concurrent peritonitis) (Fig. 1).

The 136 cases included in the study had a mean age of 34.4 years (SD: 14.5), The male-to-female ratio was 3:1. Open laparotomy was the source control procedure for all the 136 cases; 105 (77.2%) cases had laparotomy within 24 h of admission while the rest, 31 (22.8%) were operated on after 24 h of admission. Generalized peritoneal contamination occurred in 119 (87.5%) cases while 17 (12.5%) cases had localized peritoneal contamination. The commonest source of peritoneal contamination in this cohort was gastroduodenal perforation; (42.5% of cases) followed by appendiceal (17.6%) and small bowel perforations (16.2%).

The overall in-hospital mortality in this cohort of peritonitis patients was 12.5% (17 cases). Fourteen cases (10.3%) required an un-planned re-operation due to persistent intra-abdominal sepsis. Table 1 summarizes the baseline characteristics of study participants.

**Table 1:** Baseline and clinical characteristics of study participants

Parameter	Total: n = 136 (100%)	Mortality		p-value*
		No: n = 119 (87.5%)	Yes: n = 17 (12.5%)	
Mean age: years (95% CI))	34.4 (31.9–26.9)	32.9 (30.5–35.3)	45.2 (35.6–54.7)	0.002
Sex				0.335
Female	35 (25.7)	29 (82.9)	6 (17.1)	
Male	101 (74.3)	90 (89.1)	11 (10.9)	
* Comorbidities *				
Cardiovascular disease				0.106
No	134 (98.5)	118 (88.1)	16 (11.9)	
Yes	2 (1.5)	1 (50.0)	1 (50.0)	
Chronic kidney disease				0.106
No	134 (98.5)	118 (88.1)	16 (11.9)	
Yes	2 (1.5)	1 (50.0)	1 (50.0)	
Malignancy				< 0.0001
No	130 (95.6)	117 (90.0)	13 (10.0)	
Yes	6 (4.4)	2 (33.3)	4 (66.7)	
*Preoperative clinical parameters*				
sBP < 100mmHg				< 0.0001
No	129 (94.9)	119 (92.2)	10 (7.8)	
Yes	7 (5.1)	0 (0)	7 (100)	
RR > = 22 breaths/minute				0.336
No	28 (20.6)	26 (92.9)	2 (7.1)	
Yes	108 (79.4)	93 (86.1)	15 (13.9)	
SpO_2_ < 90%				< 0.0001
No	122 (89.7)	115 (94.3)	7 (5.7)	
Yes	14 (10.3)	4 (28.6)	10 (71.4)	
AVPU scale not alert				< 0.0001
No	127 (93.4)	118 (92.9)	9 (7.1)	
Yes	9 (6.6)	1 (11.1)	8 (88.9)	
Mean hemoglobin level (g/dL)	13.7 (13.2–14.2)	14.0 (13.5–14.4)	11.8 (10.6–13.1)	0.002
White blood cell count (×10^9^/L)				0.094
< 4	11 (8.1)	8 (72.7)	3 (27.3)	
4–12	78 (57.3)	72 (92.3)	6 (7.7)	
> 12	47 (34.6)	39 (83.0)	8(17.0)	
Mean platelet count (×10^3^/uL)	305.9 (283.4–328.4)	313.6 (290.3–337.0)	251.9 (174.5–329.4)	0.073
Source of peritonitis				0.005
Gastroduodenal	62 (45.6)	57 (91.9)	5 (8.1)	
Small bowel	22 (16.2)	18 (81.8)	4 (18.2)	
Appendiceal	24 (17.6)	24 (100)	0 (0)	
Colonic	12 (8.8)	8 (66.7)	4 (33.3)	
Hepatobiliary	1 (0.7)	0 (0)	1 (100)	
Genitourinary	2 (1.5)	2 (100)	0 (0)	
Others^b^	13 (9.6)	10 (76.9)	3 (23.1)	
Extend of peritonitis				0.096
Localised	17 (12.5)	17 (100)	0 (0)	
Generalised	119 (87.5)	102 (85.7)	17 (14.3)	
Reoperation				0.055
Yes	14 (10.3)	10 (71.4)	4 (28.6)	
No	122 (89.7)	109 (89.3)	13 (10.7)	

*p-value from chi-square statistics or t-test; CVD; Cardiovascular Disease; CKD: Chronic kidney disease; AVPU: Alert/Verbal/Pain/ Unresponsive consciousness scale; sBP: systolic blood pressure; n; absolute frequency; ^b^unspecified source, and pancreatitis

### Prediction of peritonitis in-hospital mortality using the PIPAS severity score and the qSOFA score tools

The mean PIPAS severity score and qSOFA score for our peritonitis cohort were 1.1 (SD: 0.9) and 0.9 (SD: 0.6) respectively. The mean PIPAS severity score for males versus females were 1.1 (SD: 0.8) and 1.2 (SD: 1.1) respectively while the corresponding male versus female mean qSOFA scores were 0.9 (SD: 0.6) and 0.9 (SD: 0.7) respectively.

Peritonitis survivors had a mean PIPAS severity score of 0.9 (SD: 05) compared to a mean PIPAS severity score of 2.8 (SD: 1.2) for non-survivors. Similarly, the mean qSOFA score for peritonitis survivors was 0.8 (SD: 0.4) versus 1.8 (SD: 1.0) for non-survivors.

Logistic regression models for peritonitis mortality using only variables of each tool were both highly statistically significant with good fits (qSOFA: Hosmer-Lemeshow chi^2^ 1.21, p = 0.997 and PIPAS: Hosmer-Lemeshow chi^2^ = 1.41, p = 0.495).

**Fig. 2:**
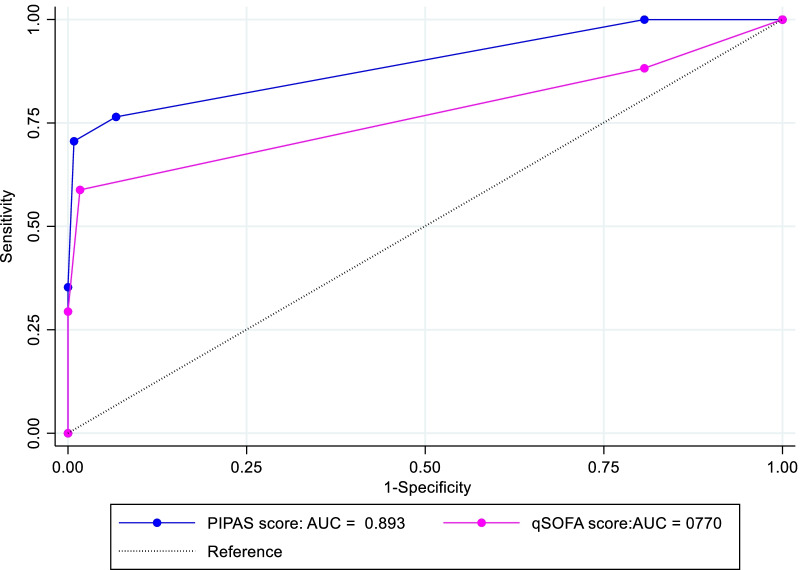
Comparative ROC curves for the PIPAS severity score and the qSOFA score tool for predicting in-hospital mortality of peritonitis

Table 2 shows the ability of the qSOFA and the PIPAS severity score tools to discriminate peritonitis survivors from non-survivors while Fig. 2 shows the comparative ROC curves. Overall, the PIPAS severity score tool had a significantly better discriminative ability for peritonitis in-hospital mortality than the qSOFA score tool (AUC = 0.893 CI 0.802–0.984) and AUC = 0.770, 95% CI (0.620–0.920) respectively, p = 0.047).

**Table 2:** Discriminative ability of the PIPAS severity score tool and the qSOFA for in-hospital mortality of peritonitis

Classifier (tool)	AUC	Bias	Bootstrap standard error	95% confidence interval
PIPAS severity score	0.893	< 0.0001	0.047	0.801–0.986 (N) 0.794–0.976 (P) 0.787–0.974 (BC)
qSOFA score	0.7701	0.00023	0.077	0.620–0.920 (N) 0.609–0.914 (P) 0.603–0.906 (BC)

AUC: Area Under the ROC Curve; N: normal confidence interval; P: percentile confidence interval; BC: Bias corrected confidence interval

The best PIPAS severity score cut-off value for predicting in-hospital mortality of peritonitis was a score of 2 or more with a sensitivity of 76.5% and a Specificity of 93.3%. Similarly, the best qSOFA score cut-off value for predicting in-hospital mortality of peritonitis was a score of 2 or more with a sensitivity and specificity of 58.8% and 98.3% respectively (Table 3).

**Table 3:** Sensitivity and specificity values for various PIPAS severity score cut-off values for predicting in-hospital mortality of peritonitis

	PIPAS severity score tool			qSOFA score tool		
Cut-off scores	Sensitivity (%)	Specificity (%)	Youden’s index	Sensitivity (%)	Specificity (%)	Youden’s index
≥ 0 score	100	0.00	0.19	100	0.00	-
≥ 1 score	100	19.3	0.19	88.2	19.3	0.08
≥ 2 scores*	76.5	93.3	0.70	58.8	98.3	0.57
≥ 3 score	70.6	99.2	0.70	29.4	100	0.29
≥ 4 score	35.3	100	0.35	-	-	-

*Optimum cut-off score value for predicting in-hospital mortality of peritonitis

## Discussion

This study compared the predictive ability of two bedside sepsis-related tools (the PIPAS severity score and the qSOFA criteria) for peritonitis in-hospital mortality in a real-time clinical setting of a low-resourced surgical emergency center. We would like to point out that, the successful application of these two tools in this study proves that both tools can be practically feasible even in a low resource setting.

The in-hospital mortality rate (12.5%) in this cohort of peritonitis patients is in the 10–20% range of peritonitis mortality generally reported in the sub-Saharan African region [25–29] but still higher than those reported in the western world [5, 30]. The most likely explanation for this is the difference in the quality of the health care system that exists between these regions; the finding from the African surgical outcome study (ASOS) alluded to this difference [31].

We found the PIPAS severity score to have a significantly superior discriminative ability for in-hospital mortality of peritonitis than the qSOFA score tool. To the best of our knowledge, this is the first time these tools have been compared however, similar trends in the discriminative ability of these tools for peritonitis mortality have been reported in a couple of non-comparative studies. Sartelli and colleagues found a good discriminative ability of the PIPAS severity score tool for mortality among a large cohort of peritonitis patients (AUC = 0.85) [10]. One other study that evaluated the predictive ability of various scoring systems for mortality of peritonitis concluded that qSOFA has a fair discriminative ability (AUC = 0.723) [22].

Furthermore, we found the PIPAS severity score tool to have a higher sensitivity than the qSOFA criteria although the latter tool is more specific. Several recent observational studies have also indicated that qSOFA criteria have low sensitivity but high specificity for identifying septic patients who are at high risk of mortality[18–22, 25]. The implication of this in practice is that the use of the qSOFA criteria as the sole initial prognostic tool for potentially septic patients would miss out on a significant number of otherwise high-risk patients for sepsis-related mortality. This is critical particularly in the management of peritonitis as the mortality associated with delayed and/or inappropriate management of these cases is extremely high [2, 10, 12].

The superiority of the PIPAS severity score tool over the qSOFA criteria both in terms of discriminative ability and sensitivity for predicting peritonitis mortality favors the former tool as the preferred initial prognostic screening tool for peritonitis.

This study had some limitations. First, it was a single tertiary center observation study. The population evaluated may be a lot sicker and biased towards poor outcomes owing to delays resulting from several levels of referral. The finding therefore may or may not be generalizable to those in lower-level surgical emergency centers. Secondly, we used qSOFA and PIPAS Severity score values that were assessed one time preoperatively to predict mortality outcomes in the study. A serial assessment including during the postoperative period would give added information including deaths attributable to postoperative complications (Additional file 1).

## Conclusions

The in-hospital mortality in this cohort of peritonitis cases was high. The PIPAS severity score tool has a superior predictive ability for in-hospital mortality of peritonitis when compared to the qSOFA score tool. The PIPAS score cut-off value of 2 or more scores has a higher sensitivity than the qSOFA criteria however, the latter tool is more specific.

We recommend the use of the PIPAS severity score tool either alone or in combination with the qSOFA criteria as the initial prognostic tool for peritonitis in the emergency department. qSOFA should not be used as a lone initial prognostic tool for peritonitis due to its low sensitivity.

## Supplementary Information

**Additional file 1: Table S1.** Study participant data used in the analysis. **Table S2.** Code labels for study participant data in the Table S1.

## Data Availability

The datasets used and/or analyzed during the current study have been attached as a supplementary file. Additional materials may be obtained from the corresponding author on a reasonable request.

## Abbreviations

A&E: Accident and Emergency
APACHE II: Acute physiological And Chronic Health Evaluation II
AUC: Area Under the Receiver Operating Characteristic curve
AVPU: Alert/ Verbal response/ response to Pain/Unresponsive scale
CI: Confidence interval
CKD: Chronic Kidney Disease
CVD: Cardiovascular Diseases
GCS: Glasgow Coma Scale
HDU: High dependence Unit
ICU: Intensive Care Unit
MNRH: Mulago National Referral Hospital
MPI: Mannheim Peritonitis Index
PIPAS: Physiological indicators for prognosis in Abdominal sepsis
PIRO-IAS: Predisposition Infection, Response Organ dysfunction score for Intra-Abdominal Sepsis
qSOFA: Quick Sequential (sepsis-related) Organ Function Assessment
ROC curve: Receiver Operating Characteristic curve
SpO_2_: Peripheral blood Oxygen saturation
SoM-REC: Makerere University School of Medicine’s Research and Ethics Committee
UNCST: Uganda National Council of Science and Technology
WSES: World Society of Emergency Surgery
WSESSSS: World Society of Emergency Surgery’s Sepsis Severity Score
