# Supplementary material for: Comparison of the PIPAS severity score tool and the QSOFA criteria for predicting in-hospital mortality of peritonitis in a tertiary hospital in Uganda: a prospective cohort study
Source: BMC Surg. 2022 Jul 28;22:291. doi: 10.1186/s12893-022-01743-4 (PMC9331107; doi:10.1186/s12893-022-01743-4)
Supplement: Supplementary file 1 — Additional file 1: Table S1. Study participant data used in the analysis. Table S2. Code labels for study participant data in the Table S1. [file 12893_2022_1743_MOESM1_ESM.docx]

**Table S1**: Study participant data used in the analysis:

| Sex (male/female) | Age category >=80 years | Malignancy (yes/no) | Severe CVD (yes/no) | Severe CKD (yes/no) | Systolic BP less than 100mmHg (yes/no) | Respiratory rate >= 22 (Yes/no) | Spo2 <90% (yes/no) | AVPU response scale not alert (yes/no) | Total PIPAS severity Score. | Total qSOFA score. | Reoperations | Outcome (survivor/non-survivor) | Extend of peritoneal Contamination (localised/ generalised) | Source of peritoneal Contamination | Preoperative white blood cell count (x10^9^) | Preoperative hemoglobin level (g/dL) | Preoperative platelet count (x10^3^) |
| --- | --- | --- | --- | --- | --- | --- | --- | --- | --- | --- | --- | --- | --- | --- | --- | --- | --- |
| 2 | 0 | 0 | 0 | 0 | 0 | 0 | 0 | 0 | 0 | 0 | 2 | 1 | 2 | 2 | 4-12 | 15.1 | 311 |
| 1 | 0 | 0 | 0 | 0 | 0 | 0 | 0 | 0 | 0 | 0 | 1 | 1 | 2 | 2 | 4-12 | 12.7 | 400 |
| 2 | 0 | 0 | 0 | 0 | 0 | 1 | 0 | 0 | 1 | 1 | 2 | 1 | 2 | 1 | 4-12 | 15.5 | 312 |
| 1 | 0 | 0 | 0 | 0 | 0 | 1 | 0 | 0 | 1 | 1 | 2 | 1 | 2 | 4 | 4-12 | 12.1 | 411 |
| 2 | 0 | 0 | 0 | 0 | 0 | 1 | 1 | 0 | 2 | 2 | 1 | 1 | 2 | 1 | >12 | 16.3 | 309 |
| 2 | 0 | 0 | 0 | 0 | 0 | 1 | 1 | 1 | 3 | 2 | 1 | 2 | 2 | 2 | >12 | 12 | 422 |
| 2 | 0 | 0 | 0 | 0 | 0 | 1 | 0 | 0 | 1 | 1 | 2 | 1 | 2 | 1 | 4-12 | 13 | 189 |
| 2 | 0 | 0 | 0 | 0 | 0 | 0 | 0 | 0 | 1 | 1 | 2 | 1 | 2 | 2 | 4-12 | 13.2 | 324 |
| 2 | 0 | 0 | 0 | 0 | 0 | 1 | 0 | 0 | 1 | 1 | 2 | 1 | 2 | 1 | 4-12 | 17.1 | 433 |
| 1 | 0 | 0 | 0 | 0 | 0 | 1 | 0 | 0 | 1 | 1 | 2 | 1 | 2 | 3 | >12 | 13.7 | 400 |
| 2 | 0 | 0 | 0 | 0 | 0 | 1 | 0 | 0 | 1 | 1 | 2 | 1 | 2 | 2 | 4-12 | 14.7 | 321 |
| 2 | 0 | 0 | 0 | 0 | 0 | 1 | 0 | 0 | 1 | 1 | 2 | 1 | 2 | 1 | 4-12 | 17.1 | 232 |
| 2 | 0 | 0 | 0 | 0 | 0 | 1 | 0 | 0 | 1 | 1 | 2 | 1 | 2 | 1 | 4-12 | 14.4 | 412 |
| 2 | 0 | 0 | 0 | 0 | 0 | 0 | 0 | 0 | 0 | 0 | 2 | 1 | 1 | 3 | 4-12 | 15.8 | 226 |
| 2 | 0 | 0 | 0 | 0 | 0 | 1 | 0 | 1 | 1 | 1 | 2 | 2 | 2 | 2 | >12 | 12.8 | 345 |
| 2 | 0 | 0 | 0 | 0 | 0 | 1 | 0 | 0 | 1 | 1 | 2 | 1 | 2 | 1 | 4-12 | 13.3 | 189 |
| 2 | 0 | 0 | 0 | 0 | 0 | 1 | 0 | 0 | 1 | 1 | 2 | 1 | 2 | 1 | 4-12 | 14.9 | 338 |
| 1 | 0 | 0 | 0 | 0 | 0 | 0 | 0 | 0 | 0 | 0 | 2 | 1 | 2 | 3 | 4-12 | 12.6 | 328 |
| 2 | 0 | 0 | 0 | 0 | 0 | 1 | 0 | 0 | 1 | 1 | 2 | 1 | 2 | 1 | 4-12 | 16 | 411 |
| 1 | 0 | 0 | 0 | 0 | 0 | 1 | 0 | 0 | 1 | 1 | 2 | 1 | 2 | 1 | 4-12 | 15 | 316 |
| 1 | 0 | 0 | 0 | 0 | 0 | 1 | 0 | 0 | 1 | 1 | 2 | 1 | 2 | 1 | >12 | 9.4 | 651 |
| 2 | 0 | 0 | 1 | 0 | 0 | 1 | 0 | 0 | 2 | 1 | 2 | 1 | 2 | 4 | 4-12 | 13.7 | 419 |
| 2 | 0 | 1 | 0 | 0 | 0 | 1 | 0 | 0 | 2 | 1 | 2 | 1 | 2 | 1 | >12 | 11.1 | 188 |
| 2 | 0 | 0 | 0 | 0 | 0 | 1 | 0 | 0 | 1 | 1 | 2 | 1 | 2 | 7 | 4-12 | 12.7 | 93 |
| 1 | 0 | 1 | 0 | 0 | 0 | 1 | 0 | 0 | 2 | 1 | 2 | 1 | 2 | 6 | >12 | 8.5 | 217 |
| 1 | 0 | 0 | 0 | 0 | 0 | 1 | 0 | 0 | 1 | 1 | 2 | 1 | 2 | 1 | 4-12 | 18.3 | 308 |
| 2 | 0 | 0 | 0 | 0 | 0 | 1 | 0 | 0 | 1 | 1 | 1 | 1 | 2 | 2 | 4-12 | 13.7 | 259 |
| 2 | 0 | 0 | 0 | 0 | 0 | 0 | 0 | 0 | 0 | 0 | 2 | 1 | 1 | 3 | >12 | 12 | 218 |
| 1 | 0 | 0 | 0 | 0 | 0 | 1 | 0 | 0 | 1 | 1 | 2 | 1 | 2 | 3 | >12 | 13.3 | 347 |
| 2 | 0 | 0 | 0 | 0 | 0 | 0 | 0 | 0 | 1 | 1 | 2 | 1 | 2 | 3 | 4-12 | 14.6 | 155 |
| 2 | 0 | 0 | 0 | 0 | 0 | 1 | 0 | 0 | 1 | 1 | 2 | 1 | 2 | 2 | 4-12 | 12.7 | 311 |
| 1 | 0 | 1 | 0 | 0 | 0 | 1 | 0 | 1 | 3 | 2 | 2 | 2 | 2 | 4 | <4 | 8 | 159 |
| 2 | 0 | 0 | 0 | 0 | 0 | 0 | 0 | 0 | 0 | 0 | 2 | 1 | 2 | 1 | >12 | 20.6 | 341 |
| 2 | 0 | 0 | 0 | 0 | 0 | 1 | 0 | 0 | 1 | 1 | 2 | 1 | 2 | 2 | 4-12 | 15.4 | 305 |
| 2 | 0 | 0 | 0 | 0 | 0 | 1 | 0 | 0 | 1 | 1 | 2 | 1 | 2 | 1 | 4-12 | 15.23 | 263 |
| 2 | 0 | 0 | 0 | 0 | 0 | 1 | 0 | 0 | 1 | 1 | 2 | 1 | 2 | 1 | <4 | 16.7 | 159 |
| 2 | 0 | 0 | 0 | 0 | 0 | 0 | 0 | 0 | 0 | 0 | 2 | 1 | 1 | 3 | >12 | 11.4 | 246 |
| 2 | 0 | 0 | 0 | 0 | 0 | 1 | 0 | 0 | 1 | 1 | 2 | 1 | 2 | 2 | 4-12 | 11.6 | 307 |
| 2 | 0 | 0 | 0 | 0 | 0 | 0 | 0 | 0 | 0 | 0 | 2 | 1 | 1 | 3 | 4-12 | 12.4 | 663 |
| 2 | 0 | 0 | 0 | 0 | 0 | 0 | 0 | 0 | 0 | 0 | 2 | 1 | 1 | 3 | 4-12 | 13.6 | 135 |
| 1 | 0 | 0 | 0 | 0 | 0 | 1 | 0 | 0 | 1 | 1 | 1 | 1 | 2 | 1 | >12 | 12.9 | 310 |
| 2 | 0 | 0 | 0 | 0 | 0 | 1 | 0 | 0 | 1 | 1 | 2 | 1 | 2 | 1 | >12 | 16.88 | 188 |
| 1 | 0 | 0 | 0 | 0 | 0 | 1 | 0 | 0 | 1 | 1 | 2 | 1 | 1 | 3 | 4-12 | 11.8 | 159 |
| 2 | 0 | 0 | 0 | 0 | 0 | 1 | 0 | 0 | 1 | 1 | 2 | 1 | 2 | 2 | 4-12 | 11.1 | 294 |
| 2 | 0 | 0 | 0 | 0 | 0 | 1 | 0 | 0 | 1 | 1 | 2 | 1 | 2 | 1 | >12 | 15.2 | 223 |
| 2 | 0 | 0 | 0 | 0 | 0 | 1 | 0 | 0 | 1 | 1 | 1 | 1 | 2 | 3 | 4-12 | 14.1 | 322 |
| 1 | 0 | 0 | 0 | 0 | 0 | 1 | 0 | 0 | 1 | 1 | 2 | 1 | 2 | 7 | >12 | 14.5 | 385 |
| 2 | 0 | 0 | 0 | 0 | 0 | 1 | 1 | 1 | 3 | 2 | 2 | 1 | 2 | 1 | >12 | 12.4 | 222 |
| 2 | 0 | 0 | 0 | 0 | 0 | 0 | 0 | 0 | 0 | 0 | 2 | 1 | 2 | 3 | 4-12 | 12.4 | 340 |
| 2 | 0 | 0 | 0 | 0 | 0 | 1 | 0 | 0 | 1 | 1 | 1 | 1 | 2 | 1 | 4-12 | 20.6 | 276 |
| 2 | 0 | 0 | 0 | 0 | 0 | 1 | 0 | 0 | 1 | 1 | 2 | 1 | 2 | 1 | 4-12 | 12.1 | 709 |
| 2 | 0 | 0 | 0 | 0 | 0 | 0 | 0 | 0 | 0 | 0 | 2 | 1 | 1 | 3 | 4-12 | 15.2 | 238 |
| 2 | 0 | 0 | 0 | 0 | 1 | 1 | 1 | 1 | 4 | 3 | 2 | 2 | 2 | 4 | >12 | 13.1 | 572 |
| 2 | 0 | 0 | 0 | 0 | 0 | 1 | 0 | 0 | 1 | 1 | 2 | 1 | 2 | 1 | 4-12 | 17.4 | 261 |
| 2 | 0 | 0 | 0 | 0 | 0 | 1 | 0 | 0 | 1 | 1 | 2 | 1 | 2 | 7 | >12 | 9.4 | 224 |
| 2 | 0 | 0 | 0 | 0 | 0 | 1 | 0 | 0 | 1 | 1 | 2 | 1 | 2 | 1 | >12 | 13.1 | 339 |
| 1 | 0 | 0 | 0 | 0 | 0 | 1 | 0 | 0 | 1 | 1 | 2 | 1 | 2 | 1 | 4-12 | 15 | 369 |
| 2 | 0 | 0 | 0 | 0 | 0 | 1 | 0 | 0 | 1 | 1 | 2 | 1 | 2 | 1 | >12 | 16.3 | 578 |
| 1 | 0 | 0 | 0 | 0 | 0 | 0 | 0 | 0 | 0 | 0 | 2 | 1 | 1 | 6 | 4-12 | 9.75 | 321 |
| 1 | 0 | 0 | 0 | 0 | 0 | 1 | 0 | 0 | 1 | 1 | 2 | 1 | 2 | 7 | 4-12 | 9.9 | 201 |
| 1 | 0 | 0 | 0 | 0 | 0 | 0 | 0 | 0 | 0 | 0 | 2 | 1 | 1 | 3 | >12 | 14.1 | 227 |
| 2 | 0 | 0 | 0 | 0 | 0 | 1 | 0 | 0 | 1 | 1 | 2 | 1 | 2 | 1 | 4-12 | 16.8 | 401 |
| 2 | 0 | 0 | 0 | 0 | 0 | 1 | 0 | 0 | 1 | 1 | 2 | 1 | 2 | 1 | 4-12 | 14.4 | 290 |
| 2 | 0 | 0 | 0 | 0 | 0 | 1 | 0 | 0 | 1 | 1 | 2 | 1 | 2 | 1 | >12 | 16 | 146 |
| 2 | 0 | 0 | 0 | 0 | 0 | 1 | 0 | 0 | 1 | 1 | 2 | 1 | 2 | 4 | >12 | 9.4 | 266 |
| 1 | 0 | 0 | 0 | 0 | 0 | 0 | 0 | 0 | 0 | 0 | 2 | 1 | 2 | 7 | >12 | 11.1 | 171 |
| 1 | 0 | 0 | 0 | 0 | 0 | 1 | 0 | 0 | 1 | 1 | 2 | 1 | 2 | 1 | 4-12 | 14.5 | 291 |
| 2 | 0 | 0 | 0 | 0 | 0 | 1 | 0 | 0 | 1 | 1 | 2 | 1 | 2 | 1 | >12 | 14.5 | 214 |
| 2 | 0 | 0 | 0 | 0 | 0 | 1 | 0 | 0 | 1 | 1 | 1 | 2 | 2 | 7 | 4-12 | 17 | 291 |
| 2 | 0 | 0 | 0 | 0 | 0 | 1 | 1 | 0 | 2 | 1 | 2 | 1 | 2 | 2 | 4-12 | 13.6 | 393 |
| 2 | 0 | 0 | 0 | 0 | 0 | 1 | 0 | 0 | 1 | 1 | 2 | 1 | 2 | 2 | 4-12 | 10.7 | 549 |
| 2 | 0 | 0 | 0 | 0 | 0 | 1 | 0 | 0 | 1 | 1 | 2 | 1 | 2 | 7 | 4-12 | 14.7 | 263 |
| 2 | 0 | 1 | 0 | 0 | 0 | 1 | 1 | 0 | 3 | 1 | 2 | 2 | 2 | 2 | 4-12 | 12.6 | 341 |
| 2 | 0 | 0 | 0 | 0 | 0 | 0 | 0 | 0 | 0 | 0 | 2 | 1 | 1 | 2 | >12 | 16.6 | 389 |
| 1 | 0 | 0 | 0 | 0 | 0 | 1 | 0 | 0 | 1 | 1 | 2 | 1 | 1 | 3 | 4-12 | 13 | 236 |
| 2 | 0 | 0 | 0 | 0 | 0 | 0 | 0 | 0 | 0 | 0 | 2 | 1 | 2 | 4 | >12 | 15.7 | 255 |
| 2 | 0 | 0 | 0 | 0 | 0 | 1 | 0 | 0 | 1 | 1 | 2 | 1 | 2 | 1 | >12 | 18 | 405 |
| 1 | 0 | 0 | 0 | 0 | 0 | 1 | 0 | 0 | 1 | 1 | 1 | 1 | 2 | 4 | >12 | 7.6 | 214 |
| 2 | 0 | 0 | 0 | 0 | 0 | 1 | 0 | 0 | 1 | 1 | 2 | 1 | 2 | 4 | 4-12 | 9.8 | 479 |
| 2 | 0 | 0 | 0 | 0 | 0 | 0 | 0 | 0 | 1 | 1 | 1 | 1 | 2 | 1 | 4-12 | 14.1 | 166 |
| 2 | 0 | 0 | 0 | 0 | 0 | 1 | 0 | 0 | 1 | 1 | 2 | 1 | 2 | 1 | 4-12 | 12 | 323 |
| 2 | 0 | 0 | 0 | 0 | 0 | 0 | 0 | 0 | 0 | 0 | 2 | 1 | 2 | 3 | 4-12 | 13.2 | 486 |
| 2 | 0 | 0 | 0 | 0 | 0 | 1 | 0 | 0 | 1 | 1 | 2 | 1 | 2 | 1 | 4-12 | 20.2 | 159 |
| 2 | 0 | 0 | 0 | 0 | 0 | 0 | 0 | 0 | 0 | 0 | 2 | 1 | 2 | 1 | >12 | 14.5 | 673 |
| 1 | 0 | 0 | 0 | 0 | 0 | 0 | 0 | 0 | 0 | 0 | 2 | 1 | 2 | 7 | 4-12 | 6.0 | 323 |
| 2 | 0 | 0 | 0 | 0 | 1 | 1 | 1 | 0 | 3 | 2 | 2 | 2 | 2 | 7 | >12 | 8.3 | 177 |
| 2 | 0 | 0 | 0 | 0 | 0 | 1 | 0 | 0 | 1 | 1 | 2 | 1 | 2 | 7 | >12 | 12.4 | 802 |
| 2 | 0 | 0 | 0 | 0 | 0 | 1 | 0 | 0 | 1 | 1 | 2 | 1 | 1 | 3 | <4 | 14.8 | 187 |
| 2 | 0 | 0 | 0 | 0 | 0 | 0 | 0 | 0 | 0 | 0 | 2 | 1 | 2 | 4 | 4-12 | 13.2 | 240 |
| 2 | 0 | 0 | 0 | 0 | 0 | 1 | 0 | 0 | 1 | 1 | 2 | 1 | 2 | 1 | 4-12 | 15.8 | 334 |
| 1 | 0 | 0 | 0 | 0 | 0 | 1 | 0 | 0 | 1 | 1 | 2 | 1 | 1 | 3 | 4-12 | 10.4 | 348 |
| 1 | 0 | 0 | 0 | 0 | 0 | 1 | 1 | 0 | 2 | 1 | 2 | 1 | 2 | 2 | <4 | 13.7 | 244 |
| 2 | 0 | 0 | 0 | 0 | 0 | 1 | 0 | 0 | 1 | 1 | 2 | 1 | 2 | 1 | >12 | 20.1 | 259 |
| 2 | 0 | 0 | 0 | 0 | 0 | 1 | 0 | 0 | 1 | 1 | 2 | 1 | 2 | 1 | <4 | 14.7 | 199 |
| 2 | 0 | 0 | 0 | 0 | 0 | 0 | 0 | 0 | 0 | 0 | 2 | 1 | 2 | 2 | 4-12 | 13.0 | 494 |
| 2 | 0 | 0 | 0 | 0 | 0 | 1 | 0 | 0 | 1 | 1 | 2 | 1 | 2 | 1 | 4-12 | 12.8 | 109 |
| 2 | 0 | 0 | 0 | 0 | 0 | 1 | 0 | 0 | 1 | 1 | 2 | 1 | 2 | 4 | 4-12 | 14.0 | 215 |
| 1 | 0 | 1 | 1 | 0 | 0 | 0 | 0 | 0 | 2 | 0 | 2 | 2 | 2 | 2 | <4 | 14.2 | 292 |
| 1 | 1 | 1 | 0 | 1 | 0 | 1 | 0 | 0 | 4 | 1 | 2 | 2 | 2 | 4 | 4-12 | 11.5 | 284 |
| 2 | 0 | 0 | 0 | 0 | 0 | 1 | 0 | 0 | 1 | 1 | 2 | 1 | 2 | 1 | 4-12 | 16.9 | 315 |
| 2 | 0 | 0 | 0 | 0 | 0 | 1 | 0 | 0 | 1 | 1 | 2 | 1 | 2 | 1 | <4 | 15.5 | 332 |
| 1 | 0 | 0 | 0 | 0 | 1 | 1 | 1 | 0 | 4 | 3 | 2 | 2 | 2 | 5 | >12 | 11.1 | 214 |
| 2 | 0 | 0 | 0 | 0 | 0 | 1 | 0 | 0 | 1 | 1 | 2 | 1 | 2 | 1 | >12 | 16.5 | 686 |
| 2 | 0 | 0 | 0 | 0 | 1 | 1 | 1 | 0 | 3 | 2 | 2 | 2 | 2 | 1 | >12 | 14.4 | 284 |
| 2 | 0 | 0 | 0 | 0 | 0 | 1 | 0 | 0 | 1 | 1 | 2 | 1 | 2 | 2 | 4-12 | 14.6 | 291 |
| 2 | 0 | 0 | 0 | 1 | 0 | 1 | 0 | 0 | 2 | 1 | 2 | 1 | 2 | 1 | 4-12 | 15.9 | 297 |
| 2 | 0 | 0 | 0 | 0 | 0 | 1 | 0 | 0 | 1 | 1 | 2 | 1 | 1 | 3 | <4 | 15.4 | 79 |
| 1 | 0 | 0 | 0 | 0 | 0 | 1 | 0 | 0 | 1 | 1 | 2 | 1 | 1 | 3 | >12 | 11.0 | 240 |
| 2 | 0 | 0 | 0 | 0 | 0 | 1 | 0 | 0 | 1 | 1 | 2 | 1 | 2 | 2 | >12 | 16.5 | 240 |
| 2 | 0 | 0 | 0 | 0 | 0 | 1 | 0 | 0 | 1 | 1 | 2 | 1 | 2 | 1 | 4-12 | 10.3 | 334 |
| 2 | 0 | 0 | 0 | 0 | 0 | 1 | 0 | 0 | 1 | 1 | 2 | 1 | 2 | 1 | 4-12 | 16.2 | 235 |
| 2 | 0 | 0 | 0 | 0 | 0 | 1 | 0 | 0 | 1 | 1 | 2 | 1 | 2 | 1 | <4 | 16.4 | 516 |
| 2 | 0 | 0 | 0 | 0 | 0 | 1 | 0 | 0 | 1 | 1 | 2 | 1 | 2 | 3 | >12 | 18.3 | 195 |
| 2 | 0 | 0 | 0 | 0 | 1 | 1 | 1 | 1 | 4 | 3 | 2 | 2 | 2 | 1 | 4-12 | 9.5 | 308 |
| 2 | 0 | 0 | 0 | 0 | 1 | 1 | 1 | 1 | 4 | 3 | 2 | 2 | 2 | 1 | >12 | 12.0 | 339 |
| 2 | 0 | 0 | 0 | 0 | 0 | 1 | 0 | 0 | 1 | 1 | 2 | 1 | 2 | 2 | 4-12 | 15.1 | 116 |
| 2 | 0 | 0 | 0 | 0 | 0 | 1 | 0 | 0 | 1 | 1 | 2 | 1 | 2 | 1 | >12 | 9.7 | 245 |
| 2 | 0 | 0 | 0 | 0 | 0 | 1 | 0 | 0 | 1 | 1 | 2 | 1 | 2 | 1 | >12 | 12.9 | 656 |
| 1 | 0 | 0 | 0 | 0 | 0 | 0 | 0 | 0 | 0 | 0 | 2 | 1 | 2 | 1 | 4-12 | 15.0 | 229 |
| 1 | 0 | 0 | 0 | 0 | 1 | 1 | 1 | 1 | 4 | 3 | 2 | 2 | 2 | 7 | >12 | 9.6 | 228 |
| 2 | 0 | 0 | 0 | 0 | 0 | 1 | 1 | 1 | 3 | 2 | 2 | 2 | 2 | 1 | 4-12 | 11.6 | 289 |
| 2 | 0 | 0 | 0 | 0 | 0 | 1 | 0 | 0 | 1 | 1 | 2 | 1 | 2 | 3 | 4-12 | 14.8 | 118 |
| 1 | 0 | 0 | 0 | 0 | 0 | 1 | 0 | 0 | 1 | 1 | 1 | 1 | 2 | 1 | <4 | 14.5 | 334 |
| 2 | 0 | 0 | 0 | 0 | 0 | 1 | 0 | 0 | 1 | 1 | 2 | 1 | 2 | 1 | 4-12 | 13.8 | 344 |
| 2 | 0 | 0 | 0 | 0 | 0 | 1 | 0 | 0 | 1 | 1 | 1 | 1 | 1 | 3 | 4-12 | 10.7 | 175 |
| 2 | 1 | 0 | 0 | 0 | 0 | 0 | 0 | 0 | 1 | 0 | 1 | 2 | 2 | 1 | <4 | 11.2 | 238 |
| 1 | 0 | 0 | 0 | 0 | 0 | 1 | 0 | 0 | 1 | 1 | 2 | 1 | 2 | 1 | 4-12 | 16.1 | 236 |
| 2 | 0 | 0 | 0 | 0 | 0 | 1 | 0 | 0 | 1 | 1 | 2 | 1 | 2 | 1 | 4-12 | 14.5 | 284 |
| 2 | 0 | 0 | 0 | 0 | 0 | 0 | 0 | 0 | 0 | 0 | 2 | 1 | 2 | 1 | 4-12 | 15.2 | Platelets |
| 2 | 0 | 0 | 0 | 0 | 0 | 1 | 0 | 0 | 1 | 1 | 2 | 1 | 2 | 2 | >12 | 13.0 | 311 |
| 2 | 0 | 0 | 0 | 0 | 0 | 1 | 0 | 0 | 1 | 1 | 2 | 1 | 2 | 1 | >12 | 16.5 | 400 |
| 2 | 0 | 0 | 0 | 0 | 0 | 1 | 0 | 0 | 1 | 1 | 2 | 1 | 2 | 7 | >12 | 12.3 | 312 |
| 2 | 0 | 0 | 0 | 0 | 0 | 1 | 0 | 0 | 1 | 1 | 2 | 1 | 2 | 1 | 4-12 | 15.4 | 411 |
| 1 | 0 | 0 | 0 | 0 | 0 | 1 | 0 | 0 | 1 | 1 | 1 | 2 | 2 | 4 | 4-12 | 12.0 | 309 |
| 1 | 0 | 0 | 0 | 0 | 0 | 1 | 0 | 0 | 1 | 1 | 2 | 1 | 2 | 7 | >12 | 9.6 | 422 |
| 1 | 0 | 0 | 0 | 0 | 0 | 1 | 0 | 0 | 1 | 1 | 2 | 1 | 1 | 3 | 4-12 | 15.9 | 189 |

*BP: Blood pressure, CKD: chronic kidney disease, CVD: cardiovascular disease, SpO2: peripheral blood oxygen saturation, qSOFA: quick sequential organ failure assessment, PIPAS: Physiologic indicators in prognosis of abdominal sepsis.*

**Table S2**: Code labels for study participant data in the Table S1.

| **Column title** | **code label** |
| --- | --- |
| Age (years) | NA |
| Sex (male/female) | 1=female, 2=male |
| Age category >=80 years | 0=No, 1=Yes |
| Malignancy (yes/no) | 0=No, 1=Yes |
| Severe CVD (yes/no) | 0=No, 1=Yes |
| Severe CKD (yes/no) | 0=No, 1=Yes |
| Systolic BP <100mmHg (yes/no) | 0=No, 1=Yes |
| Respiratory rate >=22breaths/min (yes/no) | 0=No, 1=Yes |
| SpO2 <90% at room air | 0=No, 1=Yes |
| AVPU response scale not alert (yes/no) | 0=No, 1=Yes |
| Total PIPAS severity score | NA |
| Total qSOFA score | NA |
| Reoperation (yes/no) | 1=yes, 2=no |
| Outcome (survivor/no survivor) | 1=survivor, 2=non-survivor |
| Extend of peritoneal contamination (localized/generalized) | 1=localised, 2=generalised |
| Source of peritoneal contamination | 1=Gastroduodenal, 2=small bowel, 3= large bowel, 4=appendiceal,5= hepatobiliary, 6=genitourinary, 7=others |

*BP: Blood pressure, CKD: chronic kidney disease, CVD: cardiovascular disease, SpO2: peripheral blood oxygen saturation, qSOFA: quick sequential organ failure assessment, PIPAS: Physiologic indicators in prognosis of abdominal sepsis.*
